# Burden sharing for CDR: balancing fair liability with feasibility

**DOI:** 10.1093/nsr/nwad211

**Published:** 2023-10-01

**Authors:** Chris D Jones

**Affiliations:** Met Office Hadley Centre, UK

The basics are clear: climate is changing, it is caused by human activity and it is affecting everyone and everywhere now [1]. The good news is that we know what to do about it—we can stop global temperatures rising further if we stop emitting greenhouse gases. But because some sectors (such as agriculture or aviation) will have ongoing ‘hard-to-abate’ emissions, it is also widely acknowledged that there is a requirement for some degree of carbon dioxide removal (CDR)—to bring the world to ‘net zero’. This all sounds good, but of course someone has to actually do it—but who? Past emissions have been vastly inequitable, dominated by developed countries becoming richer due to the benefits of easy and cheap energy production. So how can we equitably share the burden of reducing emissions and removing carbon?

Literature on ‘burden sharing’ for emissions reductions (e.g [2–4]) generally builds on subjective judgments around fairness such as responsibility for past emissions or current population. But to date there has been much less similar discussion on how to share the burden of the required CDR. Fyson *et al.* [5] find for example that responsibility for CDR on the basis of equity is much larger than least-cost allocation for major emitters. Pozo *et al.* [6] extend this approach to ask whether such equity-driven quotas are achievable for EU nations. Yuwono *et al.* [7] add to the debate to explain why equity definitions must be suitable for developing as well as developed countries. In a new study, Yang *et al.* [8] flesh out this discussion further and present some interesting aspects of the debate regarding the feasibility and availability of CDR at national scales.

CDR has some fundamental limits [9], not least the ability to remove CO_2_ (which typically requires large land area) and the storage potential, either in forested lands or geological reservoirs. If we expect the CDR burden to match emissions at national scales, we need to ask whether it is even possible for every country to deliver this balance. The answer according to Yang *et al.* [8] is a resounding ‘probably not’ with the size of the gap dependent on the equity assumptions. The authors hope that: ‘Identifying these gaps can help policymakers make informed decisions to address global CDR inequality.’

The novelty of Yang *et al.* [8] is to begin an assessment of balancing an equitable ‘liability’ for CDR with a ‘feasible’ delivery. Equity can never be objectively defined, so three possible definitions are compared covering financial ability to pay, population and responsibility based on past emissions. These principals, as well as the details of how to calculate them, can (and will) be hotly debated, but it is important to lay out options for discussion.

The first results that stand out are not surprising—the definition of equity can make a huge difference to the liability for each nation. For example, OECD (Organisation for Economic Co-operation and Development) countries have a much smaller liability based on current population than they do if based on past responsibility whereas less developed countries with large populations (India is the example prominently shown) have much lower responsibility-based liability than population-based ones. Liability by these definitions also differs from global scenarios that are produced by integrated assessment models based on globally cost-efficient assessment. Regions such as Latin America show up as consistently more cost-optimal (for the world) than they are liable for under equity assumptions.

Yang *et al.* [8] then go on to quantify the potential supply of CDR at the national level based on land area and geological storage. Small, densely populated, highly polluting countries such as the UK and Japan fail to have enough land. Also, large parts of Africa show up as not having sufficient geological storage. When compared with liability at a national scale, there is a global gap in terms of what CDR would be delivered. This gap grows rapidly and could be as large as 6 GtCO_2_ yr^−1^ by 2050.

Many countries have net-zero pledges or ambitions, which implicitly assumes that CDR is expected to be done on a territorial basis. But, at the national level, many countries (almost half) will be unable to deliver their equitable requirement for CDR. This leads to the inevitable conclusion of ‘an imperative demand’ for international trading separately from existing carbon markets, and for which a sound understanding of the expectations and limits of CDR at the national scale is required. Opportunities exist therefore for Latin American countries and South African countries, ‘which have abundant CDR potential after fulfilling domestic liabilities’.

This study marks an important step forward in highlighting the need to assess all aspects of CDR. Further research is also needed to better understand issues surrounding equivalence (or not) of CDR compared with simply not emitting carbon in the first place. Biophysical effects of land-based mitigation can cause unintended consequences (high-latitude reforestation, for example, has long been known to exert potentially strong warming effects [10]) and not all stores of carbon are permanent—especially if future forest stores are vulnerable to climatic extremes or increased fires or pests.

The elephant in the room is that while we are arguing over emissions reductions and what is fair, the world keeps emitting and the climate keeps warming. With every delay, not only will extremes and impacts from climate change get worse, but the solution gets harder. Current CDR activity is nowhere near what is needed and the discrepancy will grow. Yang *et al.* [8] show that a 10-year delay could nearly triple the gap between feasible and required CDR at the national level. Their study provides a big step forward in attempting to quantify these issues and guide us towards an equitable solution to a burning problem.
